# The impact of COVID-19 on people who inject drugs in New York City: increased risk and decreased access to services

**DOI:** 10.1186/s12954-021-00568-3

**Published:** 2021-11-24

**Authors:** Yesenia Aponte-Melendez, Pedro Mateu-Gelabert, Chunki Fong, Benjamin Eckhardt, Shashi Kapadia, Kristen Marks

**Affiliations:** 1 CUNY Graduate School of Public Health and Health Policy, Institute for Implementation Science in Population Health (ISPH), 55 West 125th Street, New York, NY 10027 USA; 2 NYU School of Medicine, 550 First Avenue, New York, NY 10016 USA; 3 Weill Cornell Medicine, 1305 York Ave 4th Floor, New York, NY 10021 USA; 4 NYU Rory Meyers College of Nursing, 380 Second Avenue, New York, NY 10010 USA

**Keywords:** People who inject drugs, COVID-19, HCV, New York City, Big events

## Abstract

**Background:**

While people who inject drugs (PWID) are vulnerable to the adverse outcomes of events like COVID-19, little is known regarding the impact of the current pandemic on PWID. We examine how COVID-19 has affected PWID in New York City across four domains: substance use, risk behaviors, mental health, and service utilization.

**Methods:**

As part of a randomized trial to improve access to HCV treatment for PWID, we recruited 165 participants. Eligibility criteria included detectable HCV RNA and recent drug injection. The present cross-sectional analysis is based on a subsample of 106 participants. We compared responses between two separate samples: 60 participants interviewed prior to the pandemic (pre-COVID-19 sample) and 46 participants interviewed during the pandemic (COVID-19 sample). We also assessed differences by study group [accessible care (AC) and usual care (UC)].

**Results:**

Compared to the pre-COVID-19 sample, those interviewed during COVID-19 reported higher levels of mental health issues, syringe reuse, and alcohol consumption and greater reductions in syringe-service programs and buprenorphine utilization. In the analysis conducted by study group, the UC group reported significantly higher injection risk behaviors and lower access to buprenorphine treatment during COVID-19, while during the same period, the AC group reported lower levels of substance use and injection risk behaviors.

**Conclusion:**

The current study provides insight on how COVID-19 has negatively affected PWID. Placing dispensing machines of harm-reduction supplies in communities where PWID live and increasing secondary exchange, mobile services, and mail delivery of supplies may help maintain access to lifesaving supplies during big events, such as COVID-19.

*Trial registration* ClinicalTrials.gov NCT03214679. Registered July 11 2017. https://clinicaltrials.gov/ct2/show/NCT03214679.

## Background

The novel coronavirus (COVID-19) pandemic has had a significant social, economic, and public health impact in the USA [1–4]. As of June 2021, COVID-19-related deaths have exceeded the 600,000 mark, and New York state ranks third in fatalities [5, 6]. Aside from the death toll, COVID-19 has also had a remarkable impact on every aspect of daily life. Social isolation and distancing and disruptions to health care and social services are some of the many consequences of the pandemic [7–10]. In addition, the pandemic has disproportionally impacted people from vulnerable and marginalized communities, such as people who use drugs (PWUD) [11–13]. A recent research report suggests that individuals with a history of drug use are at a higher risk for COVID-19 infection and its adverse outcomes (death and hospitalizations) than people without history of substance use [14], possibly related to pulmonary and cardiovascular medical conditions that have been associated with long-term drug use and now with COVID-19’s worst outcomes [15].

These and other medical conditions may be particularly present among people who inject drugs (PWID) [16, 17]. But PWID are also vulnerable to the worst effects of COVID-19 because, along with their complex medical comorbidities, they also experience social risk factors associated with worse pandemic outcomes [16, 17]. For instance, homelessness, poverty, marginalization, and poor access to health care have historically been linked to injection drug use and are now linked to exposure to COVID-19 [17–19]. PWID may face additional risks from COVID-19 compared to other groups because the pandemic may exacerbate preexisting challenges that many of them were already facing, such as housing and food insecurity, poor hygiene and sanitation conditions, social isolation, and stigmatization [17, 20]. Homelessness may affect some PWID’s ability to practice COVID-19 prevention measures because of a lack of access to hygiene supplies (e.g., clean water, hand sanitizer) and sanitation facilities (e.g., showers, laundry, and handwashing facilities) [17, 20]. In addition to homelessness, the need to go out to procure drugs may also increase PWID exposure to COVID-19 [15, 17]. Furthermore, previous experiences with drug-use stigmatization in health-care settings [17, 21] may influence PWID’s willingness to get tested or to seek care for COVID-19, which are imperative to controlling the spread of COVID-19. Moreover, increased drug use and drug-overdose deaths involving opioids add to negative consequences of the pandemic in the USA [22−24]. In line with trends in drug-overdose deaths nationwide, fatal and non-fatal opioid-involved overdoses have spiked in New York City, where our study was conducted, during the pandemic [23].

Emerging research from Europe, Canada, and the USA on the pandemic’s impact on PWID suggests PWID have faced considerable challenges, including loss of social connections and support, increased isolation, and deterioration of mental health as well as reduced access to social workers, counseling services, HIV/HCV testing, harm-reduction services, and inpatient drug treatment programs [9, 25–31]. Difficulties in adhering to physical distancing and complying with public health recommendations because of PWID’s living on the streets, in shelters, and in drug-treatment hostels have been reported [16, 25, 32]. In addition, the pandemic has reduced PWID’s ability to engage in income-generating activities in the formal and informal economy, making everyday life even harder [25, 27]. A study conducted in Bristol, England, found that COVID-19 has differential effects on PWID. PWID who were more stable prior to the pandemic (e.g., stable income, housing, and resources) were less vulnerable to the negative impacts of the COVID-19 lockdown and public health measures. This same study also shows how pandemic-related social isolation has exacerbated the deterioration of mental health conditions among PWID [25].

Undoubtedly, COVID-19 is what has been called a “big event” [33–36]. A big event refers to social crises triggered by diseases, hurricanes, terrorist attacks, and other phenomena that negatively affect PWID health by weakening care structures (macro-level) and changing individual behaviors (micro-level) [33–36]. At the micro-level, big events can lead to changes in drug use and injection risks [35]. At the macro-level, big events can lead to major disruptions in disease- and overdose-prevention programs [35]. The last big event in New York City was Hurricane Sandy in 2012, during which shifts in injection risk behaviors and closures of prevention services were documented [34]. For instance, PWID reported increased sharing and reusing of injection equipment after the hurricane. Pouget et al. [34] found that decreased access to substance-use treatment (methadone programs) and harm-reduction services, such as syringe-services programs (SSPs), were positively associated with increased risk behaviors. In this paper, we use a “big event” lens to explore the ways in which COVID-19 has affected PWID in New York City. To do this, we compare PWID behaviors before and during the COVID-19 pandemic to showcase the extent of the disruption the pandemic has had on their drug use, risk behaviors, mental health, and service utilization. To our knowledge, this is the first analysis that examines these domains among PWID in New York City during the pandemic.

## Methods

### Recruitment

This paper reports data from a randomized clinical trial exploring the effectiveness and feasibility of HCV care co-located in a Syringe Service Program (SSP). Print and online advertising, chain-referral sampling, street outreach, and referrals from Syringe Service Programs (SSPs) were used to recruit PWID. Eligibility criteria included the following: injected drugs in the past 90 days, 18 years or older, HCV-RNA positive, and not engaged in HCV treatment six months prior to enrollment. Individuals were pre-screened to determine eligibility. A screening visit included an assessment of drug use, HCV treatment, and HCV RNA status. Eligible participants were invited to enroll in the trial. Informed consent was obtained, and participants completed structured interviews at baseline and at three, six, nine, and 12 months. After screening and the baseline interview, participants were randomly assigned to either the accessible-care (AC) group or the usual-care (UC) group. Participants in the AC group were referred to receive low-threshold HCV care and treatment with on-site coordination at an SSP. Participants in the UC group were referred to local HCV providers with experience serving drug users. Study participants were given coupons to refer other PWID from their social networks and were compensated for completing interviews and eligible referrals. All participants were recruited and enrolled in the study before research-site closure due to COVID-19-related restrictions on March 16, 2020.

### Sample

A total of 167 participants were enrolled in the clinical trial. The present analysis is based on the latest follow-up interviews of a subsample of 106 participants, conducted from March 2019 to March 2021. This time period was selected to ensure an equal duration between the pre-COVID-19 and COVID-19 periods to assess the impact of the pandemic. We considered March 7, 2020, the beginning of the COVID-19 period, which was when New York state declared a state of emergency [37]. If a participant’s latest follow-up interview was conducted between March 29, 2019, and March 6, 2020, this interview was grouped into the pre-COVID-19 period (*n* = 60), and if the latest interview was conducted between March 7, 2020, and March 2021, it was grouped into the COVID-19 period (*n* = 46). The methodology of two independent groupings was used instead of paired grouping because of sample-size considerations and the potential for confounding participants within effect. Of the 106 interviews, one was a three-month follow-up, three were six-month follow-ups, 17 were nine-month follow-ups, and 85 were 12-month follow-ups.

The variables selected for the analysis fall into two timeframes: the past 30 days and the past 90 days. For all interviews, at least 2/3 of the timeframe inquired into occurred within the period assigned. For example, we included any participant interviewed from March 27, 2020, or later in the COVID-19 period for items that referred to the past 30 days (questions on mental health and injection risk behaviors), and we included participants interviewed from May 7, 2020, or later for items that referred to the past 90 days (questions on drug overdose, substance and alcohol use, drug-treatment programs, and SSP utilization).

### Data collection

Participant data were collected using structured interviews. These lasted between 90 and 120 min and included questions on substance use, overdose, mental health, drug injection and risk behaviors, drug-treatment programs, and SSP utilization. In addition, structured interviews included sociodemographic questions related to factors such as age, race, gender, ethnicity, housing, employment status, and HCV-treatment completion. Analyses focused on five domains: substance and alcohol use, overdose, mental health, injection risk behaviors, and drug-treatment and SSP utilization.

The interviews were conducted by trained research staff via telephone or in person. Prior to COVID-19, in-person interviews (*N* = 79) were conducted in a private room at the SSP where the study was conducted. Because of the COVID-19 pandemic, interviews from March 16 to October 2020 were completed via phone (*N* = 27) to minimize the risk of transmission and to protect participants and research staff. In the latter case, research staff mailed a mobile phone or paid for participant’s phone plans when necessary. Before the telephone interviews, research staff asked participants to find a safe and confidential place to talk. Phone interviews were scheduled in advance to further secure confidentiality and participant safety. Since study participants were enrolled prior to the closure of the study site, staff were able to maintain contact with most participants and conduct follow-up interviews during the pandemic. Participants were notified of changes in study procedures via multiple means of communication prior to the closure of the site. For example, research staff posted a sign on the door of the collaborating SSP and its mobile unit indicating changes in study operations. Participants were also contacted by phone, email, mail, and social media, as per their consent.

### Measures

In the structured interviews, homelessness was defined as staying on the streets, in a shelter, in a single-occupancy hotel, or with friends or living in a car. We assessed homelessness by the use of the terms “ever” and “90 days prior” in the interview. Employment status was defined as working full-time, working part-time, or working off-the-books jobs or disabled for work or unemployed in the past 12 months. The Addiction Severity Index was used to assess mental health status [38]. The mental health issues assessed were depression, anxiety, suicidal thoughts, suicide attempts, distress caused by emotional problems, perceived importance of getting treatment for emotional problems, and access to mental health services 30-days prior the interview. Drug use and injection were defined as using or injecting any illicit drug 90 days prior the interview. Regular drug use and injection drug use were defined as using or injecting an illicit substance three or more times a week for at least a month. The frequency of drug injection in the past 30 days was measured by the number of days a given drug was injected. Six injection risk variables were assessed: sharing syringes, cookers, cotton, water, or water containers, and using drugs that had been divided with a syringe (e.g., backloading). Sharing syringes was defined as having used a syringe that had been previously used by someone else within the 30 days preceding the interview. Sharing a cooker, water, water container, or cotton was defined as having used any of these materials simultaneously with someone else in the past 30 days or using one that had been previously used by someone else. Overdose was defined as having lost consciousness, stopped breathing, or become unresponsive as a result of illicit drug use. Questions on overdose and regular alcohol use were asked in terms of “ever” and “in the past 90 days.” Regular alcohol use was defined as using alcohol three or more times a week. Drug-treatment and SSP utilization were measured by visits to a drug treatment program or SSP within the 90 days prior the interview. HCV-treatment completion was defined as having completed antiviral therapy for HCV with direct-acting antivirals. The treatment duration is generally eight to 12 weeks, depending on medication. All measures were self-reported.

### Statistical analysis

To assess the impact of the pandemic on our sample, we first measured the differences between the pre-pandemic and pandemic cohorts for each of the variables. We then repeated this pre- and during-COVID cohort analysis separately by study group (AC and UC). Chi-square or Fisher Exact tests were used for the categorical variable depending on the sample size in the cell. Due to skewness and lack of normality, the nonparametric Mann–Whitney U test was used to compare the mean values for the continuous variables. Additionally, we measured the possible interaction between pre-COVID-19/COVID-19 and AC/UC groups by using multiple logistic regression for binary items and a generalized linear model for the skewed continuous items.

For any of the variables that presented significant results, we examined whether sociodemographic items (presented in Table 1) had confounding effects. This was done by first using bivariate analysis to determine whether there was an association between the potential confounders and the variables of interest. Second, those potential confounders presenting an association with *p* < 0.25 were entered into a multiple regression model [39]. We repeated those steps to identify possible sociodemographic confounding effects within the separated study-group (AC and UC) analyses. SPSS version 25 was used to conduct the analysis.

**Table 1:** Sample characteristics

	Overall	Period		Group condition	
		Pre-COVID	COVID	Usual care	Accessible care
	(*n* = 106)	(*n* = 60; 56.6%)	(*n* = 46; 43.4%)	(*n* = 52; 49.1%)	(*n* = 54; 50.9%)
	*n* (%)	*n* (%)	*n* (%)	*n* (%)	*n* (%)
Age					
18–29	10 (9.5)	3 (5.0)	7 (15.2)	4 (7.7)	6 (11.1)
30–39	35 (33.3)	19 (31.7)	16 (34.8)	19 (36.5)	16 (29.6)
40–49	29 (27.4)	18 (30.0)	11 (23.9)	15 (28.8)	14 (25.9)
> 50	32 (30.2)	20 (33.3)	12 (26.1)	14 (26.9)	18 (33.3)
Gender					
Male	86 (81.1)	50 (83.3)	36 (78.3)	43 (82.7)	43 (79.6)
Female	19 (17.9)	10 (16.7)	9 (19.6)	9 (17.3)	10 (18.5)
Transgender	1 (0.9)	0 (0.0)	1 (2.2)	0 (0.0)	1 (1.9)
Race/ethnicity					
Hispanic	66 (62.3)	39 (65.0)	27 (58.7)	35 (67.3)	31 (57.4)
Non-Hispanic White	28 (26.4)	12 (20.0)	16 (34.8)	13 (25.0)	15 (27.8)
Non-Hispanic Black	5 (4.7)	4 (6.7)	1 (2.2)	1 (1.9)	4 (7.4)
Other	7 (6.6)	5 (8.3)	2 (4.3)	3 (5.8)	4 (7.4)
Homelessness					
Lifetime	95 (89.6)	52 (86.7)	43 (93.5)	45 (86.5)	50 (92.6)
Past 3 months	53 (50.0)	31 (51.7)	22 (47.8)	26 (50.0)	27 (50.0)
Employment status					
Employed full time on the books	5 (4.7)	3 (5.0)	2 (4.3)	3 (5.8)	2 (3.7)
Employed part time on the books	2 (1.9)	2 (3.3)	0 (0.0)	1 (1.9)	1 (1.9)
Odd jobs, off the books	11 (10.4)	7 (11.7)	4 (8.7)	5 (9.6)	6 (11.1)
Disabled for work	12 (11.3)	8 (13.3)	4 (8.7)	7 (13.5)	5 (9.3)
Unemployed	74 (69.8)	39 (65.0)	35 (76.1)	35 (67.3)	39 (72.2)
Other	2 (1.9)	1 (1.7)	1 (2.2)	1 (1.9)	1 (1.9)
Income (past 12 month)					
$0–$10,000	62 (62.0)	35 (62.5)	27 (61.4)	30 (61.2)	32 (62.7)
$11,000–$25,000	22 (22.0)	13 (23.2)	9 (20.5)	13 (26.5)	9 (17.6)
$26,000 or greater	16 (16.0)	8 (14.3)	8 (18.2)	6 (12.2)	10 (19.6)
Participants completed treatment	55 (51.9%)	34 (56.7%)	21 (45.7%)	14 (26.9%)	41 (75.9%)**

**p < .01

## Results

### Sample characteristics

Sociodemographic characteristics are presented in Table 1. A total of 167 participants were enrolled in the study. The sample for the analysis consisted of 106 PWID who were interviewed over a two-year period (from March 2019 to March 2021). As Table 1 shows, the sample was predominantly male (81.1%). Overall, participants reported being either non-Hispanic White (26.4%), non-Hispanic Black (4.7%), or Other (6.6%). Hispanic ethnicity was reported by more than half (62.3%) of the sample. The mean age was 43.1. Half of the participants were recently homeless (50.0%), and most had incomes below $11,000 (62%) per year at the moment of study enrollment. Overall, the majority were unemployed (69.8%). Only 17% had some form of employment (either full-time, part-time, or off the books).

### Mental health

As presented in Table 2, higher levels of mental health problems were found among participants interviewed during the COVID-19 period than among those interviewed pre-COVID-19. The results show that 80.4% of participants reported psychological or emotional problems during COVID-19, a significant difference to the 50% reported before the pandemic (*p* < 0.01). Similarly, levels of depression and anxiety within the 30 days prior to the interviews were significantly higher among participants interviewed during the pandemic than those interviewed before the pandemic: 52.5% reported depression symptoms prior to the pandemic, and 84.8% (*p* < 0.01) reported symptoms during the pandemic. Symptoms of anxiety disorders were also significantly higher: 84.8% reported anxiety during the pandemic, and 49.2% (*p* < 0.01) reported anxiety in the pre-COVID-19 period. However, participants in the UC group were significantly more likely to report being troubled by psychological or emotional problems and considering important treatment for psychological problems than those in the AC group. Sociodemographic variables (presented in Table 1) did not have any confounding effects on mental health.

**Table 2:** Comparison of the 30-days items between pre-COVID and COVID periods

	Total		Usual care		Accessible care	
	Pre-COVID	COVID	Pre-COVID	COVID	Pre-COVID	COVID
	(*n* = 60; 56.6%	(*n* = 46; 43.3%)	(*n* = 30; 57.7%)	(*n* = 22; 42.3%)	(*n* = 30; 55.6%)	(*n* = 24; 44.4%)
	%	%	%	%	%	%
Mental health						
Experienced serious depression	52.5	84.8**	58.6	90.9*	46.7	79.2*
Experienced serious anxiety or tension?	49.2	84.8**	48.3	90.9**	50.0	79.2*
Experienced serious thoughts or suicide?	5.1	13.0	3.4	18.2	6.7	8.3
Attempted suicide?	1.7	2.2	0.0	4.5	3.3	0.0
In the past 30 have you experienced any psychological or emotional problems?	50.0	80.4**	53.3	81.8*	46.7	79.2*
How much have you been troubled or bothered by these psychological or emotional problems in the past 30 days? (Considerably/Extremely)	37.3	66.7**	34.6	84.2**	40.0	52.2
How important to you now is treatment for these psychological problems? (Considerably/Extremely)	37.3	57.1^	30.8	68.4*	44.0	47.8

	Mean (std)	Mean (std)	Mean (std)	Mean (std)	Mean (std)	Mean (std)
Injection frequency and risk behavior						
In the past 30 days, how many days have you injected drugs? (mean)	9.3 (12.6)	9.3 (13.0)	10.2 (13.5)	16.0 (14.9)	8.5 (11.9)	3.2 (7.0)^
On the days that you injected, on average how many times a day did you inject? (mean)	1.9 (3.3)	1.3 (2.4)	1.6 (2.4)	1.6 (1.7)	2.1 (4.1)	1.1 (2.9)^
In the past 30 days when you injected, how many times did you use a new, sterile syringe? (mean)	32.9 (69.5)	15.6 (31.6)	35.6 (69.7)	28.6 (41.3)	30.1 (70.3)	3.0 (5.9)*

	%	%	%	%	%	%
In the past 30 days when you injected, did you use a new, sterile syringe?	55.0	45.7	56.7	63.6	53.3	29.2^
In the past 30 days, did you reuse your own syringes?	8.3	19.6^	6.7	31.8*	10.0	8.3
In the past 30 days, did you give your used syringe for other to use?	6.7	10.9	6.7	18.2	6.7	4.2
In the past 30 days, did you use a syringe that had already been used by somebody else?	3.3	0.0	0.0	0.0	6.7	0.0
In the past 30 days, how many times did you share cookers?	15.0	21.7	16.7	45.5*	13.3	0.0
In the past 30 days, did you share cottons/filters	15.0	21.7	16.7	45.5*	13.3	0.0
In the past 30 days, did you share drug diluting water?	15.0	21.7	16.7	45.5*	13.8	0.0
In the past 30 days, did you share a water container?	15.0	21.7	16.7	45.5*	13.8	0.0
In the past 30 days, did you use drugs that had been divided with a syringe (e.g., backloading)?	16.7	17.4	16.7	36.4	16.7	0.0^

^ *p* < .10; * *p* < .05; ** *p* < .01

### Injection frequency and risk behaviors

Table 2 presents data showing that syringe reuse was higher among participants interviewed during the pandemic than those interviewed prior to the pandemic (8.3% vs 19.6%; *p* = 0.090). The AC group presented a marginally significant lower mean of days injecting drugs (8.5 vs 3.2; *p* = 0.059), a lower number of times injected on days when injected (2.1 vs 1.1; *p* = 0.099), and a lower percentage of participants backloading (16.7% vs 0%; *p* = 0.059) for the 30 days prior to the interview. The number of times that a new sterile syringe in the past 30 days was used was significantly lower among participants in the AC group (30.1 vs 3.0; *p* = 0.033). This decline reflects a parallel decline in the number of days and times they injected. Unlike the AC group, injection risk behaviors were significantly higher among participants in the UC group during the pandemic, and they were more likely to report sharing injection equipment. A significantly higher percentage of participants in UC group (16.7% prior the pandemic, compared to 45.5% during the pandemic; *p* = 0.024) reported sharing cookers, water, water containers, and cotton during the COVID-19 period. Participants in this group were also more likely to report reusing syringes (6.7% vs 31.8%; *p* = 0.027) than their AC counterparts. No sociodemographic confounding effects were found for injection frequency and injection risk behaviors.

### Overdose and substance use

Overall, there were no significant changes in substance use (Table 3). However, there was a drop in drug use among participants in the AC group during the pandemic. Reduction in drug use was associated with the AC group. While the percentage of participants who used drugs in the past 90 days were lower in the AC group (70% during COVID-19 period vs 93.1% pre-COVID-19; *p* = 0.0497), there were no significant changes in drug use for those in the UC group. This significance remained once we checked for possible confounders. While, overall, regular alcohol use was significantly higher during the pandemic (29.7% vs 12.5%; *p* = 0.040), participants in the UC group were more likely to drink regularly than those in the AC group. The rise in regular alcohol use was confounded by gender and lifetime homelessness.

**Table 3:** Comparison of the 90-days items between Pre-COVID and COVID Periods

	Total		Usual Care		Accessible Care	
	Pre-COVID	COVID	Pre-COVID	COVID	Pre-COVID	COVID
	(*n* = 56; 60.2%)	(*n* = 37; 39.8%)	(*n* = 27; 61.4%)	(*n* = 17; 38.6%)	(*n* = 29; 59.2%)	(*n* = 20; 40.8%)
	%	%	%	%	%	%
Overdose						
Have you overdosed in the past 90 days?	5.4	13.5	3.7	5.9	6.9	20.0
Emergency Personnel Have you had emergency personnel come because of an overdose in the past 90 days?	0.0	5.4	0.0	0.0	0.0	10.0
Do you currently have Narcan/Naloxone (e.g., at your house, in your purse)?	32.1	24.3	33.3	47.1	31.0	5.0*
Substance use						
Any drug use past 90 days	83.9	70.3	74.1	70.6	93.1	70.0*
Any regular drug use past 90 days	62.5	54.1	59.3	58.8	65.5	50.0
Any drug injection past 90 days	62.5	48.6	55.6	52.9	69.0	45.0^
Any regular drug injection past 90 days	42.9	35.1	40.7	47.1	44.8	25.0
Have you drunk alcohol in the past 90 days?	19.6	29.7	22.2	35.3	17.2	25.0
Have you drunk alcohol regularly in the past 90 days? (Regular use = 3 or more times/week)	12.5	29.7*	11.1	35.3^	13.8	25.0
Drug treatment						
Have you been in treatment for drug use in the past 90 days?	51.8	54.1	44.4	47.1	58.6	60.0
Buprenorphine treatment alone—drug treatment in the past 90 days	8.9	2.7	3.7	5.9	13.8	0.0
Buprenorphine treatment plus outpatient counseling—drug treatment in the past 90 days	17.9	5.4	25.9	0.0*	10.3	10.0
Methadone maintenance program—drug treatment in the past 90 day	35.7	51.4	33.3	41.2	37.9	60.0
Residential detoxification program (< = 1wk)—drug treatment in the past 90 days	1.8	0.0	0.0	0.0	3.4	0.0
Residential rehabilitation program (28-day program)—drug treatment in the past 90 days	0.0	10.8*	0.0	5.9	0.0	15.0^
Long term residential treatment—drug treatment in the past 90 days	3.6	2.7	7.4	0.0	0.0	5.0
Regular 12-step attendance—drug treatment in the past 90 days	1.8	0.0	3.7	0.0	0.0	0.0
Syringe services program						
Have you been to the needle exchange in the past 90 days?	44.6	27.0^	48.1	41.2	41.4	15.0^

^ *p* < .10; *p < .05

Overall, there was no significant change in overdose events from the pre-COVID-19 to the COVID-19 period. In addition, naloxone possession was significantly lower during the pandemic among those in the AC group (from 31% down to 5%), although age was identified as a confounder for this effect.

### Drug treatment and syringe-services programs

There was a decline in some drug-services utilization from the pre-COVID-19 to the COVID-19 period (Table 3). Specifically, COVID-19 appears to have marginally affected the utilization of SSPs overall (44.6% pre-COVID-19 to 27% during COVID-19; *p* = 0.086) and significantly affected buprenorphine treatment in the UC group (25.9% pre-COVID-19 versus 0% during COVID-19; *p* = 0.032). Overall, between periods, there were no significant changes in the utilization of methadone-maintenance programs. However, participants were more likely to report utilizing 28-day in-patient programs (0% pre-COVID-19 vs 10.8% during COVID-19; *p* = 0.023) during the pandemic. No confounding factors were found for drug-treatment and syringe-exchange utilization.

## Discussion

From the pre-pandemic to the pandemic period, the overall study sample reported higher levels of mental health problems, need for psychological treatment, syringe reuse, and alcohol consumption. In addition, a decline in SSP and buprenorphine utilization were observed.

However, differences were identified between study groups as regards drug-use behaviors and service utilization during the COVID-19 period. For instance, levels of alcohol use and injection risk behaviors were higher among those in the UC group. The UC group also reported a significant reduction in access to buprenorphine treatment with outpatient counseling. The AC group reported a significant decline in substance use and average injections per day and a steep increase in enrollment in 28-day drug programs. Engagement in injection risk behaviors was associated with the UC group, while disengagement in these same measures were identified among those in the AC group. A plausible explanation for the lower levels of drug use and injection risk behaviors in the latter group is the reception of HCV treatment and a re-infection prevention intervention, which would have occurred between the pre-COVID-19 period and the COVID-19 period. In this study, the AC group had a higher portion of participants who completed the HCV treatment when compared to the UC group (75.9% versus 26.9%; *p* < 0.01; Table 1). Hence, our results suggest that treatment completion and educational intervention may have attenuated the association relationship between COVID-19 and higher injection risk behaviors. Other authors have also reported that completion of HCV treatment is associated with reduction in HCV-related risk behaviors [40].

Across study groups, access to methadone-program services did not change. While the previous big event in New York, Hurricane Sandy, caused major interruptions and closures of New York City methadone programs [41], our findings suggest that COVID-19 did not trigger such disruptions among our participants. This might be due to policy changes implemented during the pandemic. In an effort to maintain access to methadone while preventing the spread of COVID-19, the US federal government altered methadone-dispensing regulations to increase access to “take-home doses” of methadone and temporarily waived the daily in-person visits requirement [42]. The implementation of this flexible methadone-dosing system in New York City may have helped PWID on methadone avoid treatment discontinuation due to COVID-19 [43]. This policy change may have improved access to methadone for those in this study. This finding is congruent with data that show that flexibility in methadone dispensation during the pandemic has been an effective way to ensure the continuity of methadone treatment for people with opioid-related problems [44, 45]. Interestingly, while the federal government also loosened regulations around buprenorphine [46, 47], a decrease in access to buprenorphine (especially with outpatient counseling) was observed among PWID in this study. However, regulatory changes allowed buprenorphine access care via telehealth, and it is possible that telehealth visits were not feasible for our study population. Most of our participants were older, poor, and homeless, and they lacked access to the technology necessary for telemedicine (smartphones, computers). Undoubtedly, more research is needed to explore buprenorphine access during the pandemic.

The most prominent of the pandemic’s effects on both groups was its impact on mental health problems, which increased across groups. This finding is consistent with research studies that found higher rates of mental health issues during the pandemic in both the general population [48–50] and people who use drugs [25, 27, 51]. It is possible that social restrictions (prolonged lockdowns, isolation) and social services disruptions during the pandemic have affected participants’ sense of social connection and increased their levels of depression, anxiety, and suicidal thoughts. Before the pandemic, participants were among the most marginalized and stigmatized members of society, and the pandemic may have increased their marginalization. For instance, many attended SSPs regularly prior the pandemic and, in many cases, spent a large part of their day at these programs. SSPs not only provide safer drug-use supplies, food, counseling, and other services but also play an important role in participants’ everyday social lives. SSPs are important components of social integration for PWID. According to sociologist Emile Durkheim, individuals’ integration into social institutions, such as community-based organizations, can reduce alienation from society by increasing social ties [52]. Strong ties with community organizations, such as SSPs, can increase access to resources that they could not otherwise access [30]. These programs are social settings where participants have face-to-face interactions and develop systems of social and emotional support [30]. Participation in these types of programs may be beneficial for their mental health since it enhances opportunities for social interaction, reduces social isolation, and provides social and emotional support [30, 53, 54]. These crucial social ties and system of support are precisely the ones that have been altered during the pandemic, and we believe help explain this important finding regarding steep mental health deterioration. Our findings mirror previous studies that have shown that a reduction in social interactions/networks during big events can lead to negative mental health consequences [55–58].

Although many SSPs have reopened at the time of writing, face-to-face activities and drop-in centers remain limited. These reductions in access to SSPs help explain the sharp decline in SSP use and naloxone possession among study participants. These findings regarding reduced access to SSPs align with existing data that indicate a decline in the availability and provision of these services in Europe, Latin America, and the USA [9, 15, 59]. Several studies have documented a reduction in service utilization, as well as operational changes such as full or partial suspension of HIV/HCV testing, naloxone training and distribution, aid groups, drop-in centers, and medical and counseling services, particularly at the initial stages of the pandemic [9, 15, 59]. Furthermore, data from an international survey show that SSPs have been among the most affected globally compared to drug treatment services (i.e., methadone, buprenorphine), especially in low-income countries [59].

It is worth noting that many SSPs have adapted by replacing on-site services with telephone and video calls, doorstep delivery, and mobile services [25, 60, 61]. Despite these changes to support PWID, SSPs have experienced significant service disruptions since the arrival of COVID-19. Barriers to accessing technology-based services and support on the part of PWIDs have been reported by Kesten et al. in their study on the impact of COVID-19 among PWID in Bristol [25]. Kesten et al. reported that the lack of access to telephones and an adequate internet connection were identified by some PWID as barriers to access technology-based services implemented by SSPs in the COVID-19 era. In addition, the impersonal nature of connecting and meeting with others online were noted as challenges for some PWID. The authors also reported participants concern regarding loss of privacy and confidentiality when receiving doorstep delivery services.

As occurred during Hurricane Sandy’s aftermath in New York, the decline in SSPs may have led to an overall significant increase in needle reuse and an increase in injection-equipment sharing in the UC group. Nine years after Hurricane Sandy, yet another big event has disrupted SSPs, leaving marginalized PWID without lifesaving services. It is imperative that, as Pouget and colleagues [34] suggested after Hurricane Sandy, local and state agencies work in collaboration with harm-reduction organizations, such as SSPs, to develop plans in advance to reduce the harm that big events can have on PWID, especially since these events are becoming more frequent [35]. For example, state authorities can allow and fund harm-reduction dispensing machines to provide free injection equipment and naloxone in places where PWID congregate, such as near local grocery stores (“bodegas”), drug spots, community centers, and homeless shelters, to mitigate the risks the pandemic forced upon PWID. Indeed, having dispensing machines in communities where PWID live and spend most of their time may be a valuable and effective tool to improve and expand access to services without in-person interactions. This is particularly true for homeless PWID. These machines would not only provide PWID access to lifesaving supplies but would allow participants to access services without disclosing their drug-use status or feeling shame or stigma. In addition to implementing dispensing machines, increasing and expanding secondary exchanges [61], mobile syringe-exchange services [25], and free mail delivery of harm-reduction supplies [62] can also help PWID during big events. The incorporation of an “on-site telephone-booth” model that emerged in California during the pandemic may be another effective way to reduce some of the obstacles encountered by some PWID, particularly the homeless [63]. The on-site telephone booth allows SSP participants who lack technological tools or prefer in-person care to access care and communicate with social and medical providers on-site while complying with physical-distancing requirements. While it has been reported that fatal and non-fatal opioid-related overdoses have increased during the pandemic [23], we did not observe significant changes in drug overdoses in our study. This finding may be due to the small sample size. It is also possible, however, that the intervention had an overdose-protective effect for those in the AC group. Because PWID in the AC group reduced substance use during the pandemic, maintained methadone treatment, and even increased enrollment in drug treatment (i.e., 28-day programs), their overdose risks may have been mitigated. While this only applies to those in the AC group and not those in the UC group, it may still help explain the lack of change in overdose events due to COVID-19 in this study. More research is needed to explain this finding.

There are several limitations that should be noted. First, the clinical trial and survey questions were not designed to assess the impact of COVID-19; hence, we cannot determine whether the results presented are a consequence of the pandemic or would have occurred as a result of the trial about improving access and completion of HCV treatment. Given the nature of the cross-sectional design of this analysis, we must also exercise caution ascribing differences in drug use and injection risk behaviors between study groups during the pandemic to HCV treatment. Similarly, we cannot be certain that changes in mental health status were directly related to COVID-19. Second, the sample used in this study is small and is limited by the exclusive enrollment of HCV-positive PWID in New York City. Therefore, findings may not be generalized to all PWID in New York and the USA. In addition, the majority of the participants were male, which limits the study’s generalizability to women who use drugs. Future studies are needed to understand the short- and long-term impact of COVID-19 on drug users from different geographical areas and with differing HCV statuses. Third, this analysis is based on self-reported data, which is susceptible to recall and desirability bias. Data are dependent on the memory and honesty of participants. We trained interviewers in being non-judgmental and providing a comfortable environment, so the participants would be more likely to give an honest response. Despite these limitations, this study begins to shed light on the potential impact of COVID-19 as a big event on PWID’s disease and overdose risks and mental health.

## Conclusions

As the pandemic continues to evolve, it is important to continue exploring the potential impact of COVID-19 among PWID, particularly in relation to mental health. Findings from this study suggest that more research might be useful in guiding public health efforts to increase access to mental health services for PWID during big events, such as COVID-19. These public efforts need to expand on existing telemedicine (virtual mental health visits) services because many PWID cannot benefit from it. PWID often lack the tools (smartphones, computers, internet access, etc.) needed to access these types of virtual services. This expansion is necessary to address inequitable access to care during big events. In addition, public efforts should include expanding syringe access through multiple innovative approaches, such as mobile syringe-exchange services, mail delivery of harm-reduction supplies, and the placement of dispensing machines in communities where PWID reside to expand access to disease- and overdose-prevention supplies and to mitigate stigma.

## Data Availability

The datasets used and/or analyzed during the current study are available from the corresponding author on reasonable request.

## Abbreviations

PWUD: People who use drugs
PWID: People who inject drugs
SSPs: Syringe services programs
SSP: Syringe services program
AC: Accessible care
UC: Usual care
HCV: Hepatitis C
HIV: Human immunodeficiency virus
